# Potential renoprotective effects and possible underlying mechanisms of angiotensin receptor-neprilysin inhibitors in cardiorenal syndrome

**DOI:** 10.3389/fmed.2024.1451450

**Published:** 2025-01-07

**Authors:** Md. Moshiur Rahman, Asadur Rahman, Akira Nishiyama

**Affiliations:** ^1^Department of Pharmacology, Faculty of Medicine, Kagawa University, Takamatsu, Japan; ^2^Department of Pharmacology and Toxicology, Faculty of Animal Science and Veterinary Medicine, Sher-e-Bangla Agricultural University, Dhaka, Bangladesh

**Keywords:** ARNIs, cardiorenal syndrome, renoprotective effects, molecular mechanisms, pre-clinical evidence

## Abstract

Angiotensin receptor-neprilysin inhibitors (ARNIs) represent a novel class of medications characterized by their dual action on major cardiorenal regulators, specifically the renin–angiotensin system (RAS) and the natriuretic peptide (NP) system. Sacubitril/valsartan, a pioneering ARNI, has demonstrated strong antihypertensive effect as well as superior efficacy in preserving renal function compared to RAS inhibitors in heart failure patients with reduced ejection fraction. Here, we gathered evidence on the impact of sacubitril/valsartan on the preservation of kidney function in patients with cardiorenal syndrome (CRS). In particular, we present a comprehensive summary of the latest advancements and findings from clinical trials, studies, and meta-analyses on the impact of ARNIs in maintaining or improving renal function. We also discussed the pre-clinical evidence supporting the use of sacubitril/valsartan for improving renal function, along with the underlying molecular mechanisms in animal models mimicking various clinical scenarios. Altogether, the analysis of published data from both pre-clinical and clinical studies provides substantial support for the usefulness of ARNIs in enhancing renal protection in subjects with CRS.

## Introduction

Cardiorenal syndromes (CRS) refer to a group of conditions wherein dysfunction in either the heart or kidneys leads to dysfunction in the other organ (1). If not managed properly, this reciprocal interaction can create a vicious cycle of deteriorating organ function. The primary goals of cardiorenal therapeutic intervention are to improve both cardiac and renal function, decelerate disease progression, reduce morbidity and mortality, and improve quality of life in patients with CRS. Hypertension often coexists with CRS, and it is crucial to achieve effective blood pressure (BP) management in order to prevent further organ damage (2). Conventional medications including angiotensin-converting enzyme inhibitors (ACEi), angiotensin II receptor blockers (ARB), and β-blockers have been usually prescribed to manage hypertension as well as cardiorenal outcomes. In recent years, the combination of angiotensin receptor as well as neprilysin inhibitors (ARNIs) has emerged as a promising therapeutic strategy for managing CRS. ARNIs offer a comprehensive approach for managing conditions that impact both the heart and kidneys by simultaneously inhibiting renin angiotensin system (RAS) and neprilysin, aiming to improve the outcomes and quality of life. To date, the majority of clinical trials and studies involving ARNIs have predominantly focused on heart failure (HF), with a secondary emphasis on assessing renal function (3–5). ARNIs, however, might have the potential to be an advantageous treatment option for patients with chronic kidney diseases (CKD), particularly those with concurrent HF, owing to their ability to diminish proteinuria and halt the decline of estimated glomerular filtration rate (eGFR) (6, 7).

## Historical development of ARNIs

Neprilysin, a neutral endopeptidase (NEP), is a key enzyme responsible for degrading natriuretic peptides (NPs) (8) and various other vasoactive peptides, including angiotensin (Ang)-I, adrenomedullin (ADM), bradykinin, neurokinin A, neuropeptide Y, substance P, and endothelin (ET)-1 (9–14). NEP is a zinc-containing membrane-bound metalloproteinase which is expressed in different tissues, such as the brain, smooth muscle, cardiac myocytes, vascular endothelial cells, and neutrophils. However, it is most abundant in the brush border of proximal renal tubular cells (9, 15). Neprilysin inhibition significantly reduced intraglomerular pressure and proteinuria in the kidney by causing natriuresis and vasodilation (16, 17). In addition, Abe et al. showed that the intrarenal infusion of synthetic human atrial natriuretic peptide led to a substantial rise in renal blood flow, urine flow, and urinary sodium excretion. This improvement in renal hemodynamics occurred without any alteration in renal perfusion pressure (18). Therefore, several pharmaceutical approaches have been studied to preserve or enhance the levels of NPs, with the aim of counteracting the excessive activation of the RAS and/or sympathetic nervous system (SNS) in cardiovascular disorders. These consist of the application of exogenous NPs (e.g., nesiritide and carperitide) and inhibiting the breakdown of NP by the use of neprilysin inhibitors (NEPIs) (e.g., racecadotril, candoxatrilat, ecadotril, candoxatril or sacubitril) (19). Accordingly, candoxatril; the first selective NEPI employed in humans, effectively elevated NP levels and reduced BP (20, 21). Furthermore, the administration of sacubitril in hypertensive rats resulted in an increase in renal hypertrophy and glomerular lesions (22). Due to the diverse array of neprilysin substrates, such as Ang II, the activity of the RAS could be elevated. This suggests that neprilysin inhibition enhances RAS activity, thereby undermining the anticipated benefits of the medication in managing hypertension and the progression of renal diseases (23). Consequently, combining NEPI with RAS blockers, particularly angiotensin converting enzyme inhibitors (ACEi), was proposed. Omapatrilat, an ARNI comprising ACEi, was extensively researched and found to have notable inhibitory effects on ACE, leading to a decrease in Ang II, as well as a reduction in systemic BP in healthy volunteers (24–26). Additionally, patients with systolic hypertension responded considerably better to omapatrilat compared to ACEi alone in terms of systolic BP and pulse pressure reduction (27, 28). Nevertheless, the combination of ACEi and NEPIs enhanced the ACEi-induced accumulation of bradykinin by inhibiting the breakdown of bradykinin through neprilysin (19), and increased the risk of angioedema (29). Therefore, the development of omapatrilat was discontinued due to incidence of severe angioedema (30). ARBs are less likely to cause angioedema since they have limited effects on bradykinin but similar cardiorenal activities as ACEi (31, 32). Eventually, ARNIs emerged as a new category of medications by combining both NEPI and ARBs without the risk of angioedema (33).

## Pharmacological effects of sacubitril/valsartan

### Effect of sacubitril/valsartan on BP

Prior preclinical studies have shown superior BP lowering effect of sacubitril/valsartan compared to ARB (e.g., valsartan) alone in different animal models (Table 1). Uijl et al. experimented on streptozotocin induced diabetic TGR (mREN2)27 rats (a model of angiotensin II-dependent hypertension) with sacubitril/valsartan for 3 weeks and found that sacubitril/valsartan significantly reduced mean arterial pressure (MAP) compared to vehicle (34). Similar BP lowering effect had been described by Ushijima et al. in a rat model of subtotal nephrectomy, where LCZ696 (sacubitril/valsartan) and valsartan significantly decreased systolic BP, but systolic BP in both (sacubitril/valsartan) and valsartan-treated groups was still higher compared to the sham-operated group (35). A separate animal study involving stroke-prone spontaneously hypertensive (SHRSP) rats observed a reduction in BP in those treated with sacubitril/valsartan or valsartan alone, compared to rats fed a Japanese diet (JD). However, significant reduction in BP was achieved in sacubitril/valsartan group by the third week of the experiment, and it remained consistently lower throughout the study period (36). To quantify the optimum dosage of the AT1 receptor blocker together with an NEPI (ARNI) that may yield beneficial effects, Roksnoer et al. conducted a comparative analysis utilizing single NEP inhibition (thiorphan) and AT1 receptor blockade (irbesartan) against the ARNI approach (thiorphan + irbesartan), applying both a low and a high thiorphan dose. The study was carried out in heterozygous TGR (mREN2)27 (Ren2) rats and they concluded neither vehicle nor thiorphan alone affected MAP. However, irbesartan, either alone or in combination with the low or high dose of thiorphan, markedly reduced MAP compared to the vehicle (37). Moreover, using OLETF rats, we found that LCZ696 had greater and significant hypotensive effect compared to valsartan alone (38). Habibi et al. determined that treatment with sacubitril/valsartan, valsartan or hydralazine for 10 weeks in Zucker obese (ZO) rats tended to lower MAP by 4.2% in sacubitril/valsartan, 3.9% in valsartan and 3.7% in hydralazine group. However, statistical significance was found only in sacubitril/valsartan by the end of the study during both light and dark cycles (39). Hypotensive effect of sacubitril/valsartan was also found in subtotal nephrectomized rats treated with either valsartan or sacubitril/valsartan by oral gavage for 8 weeks. Both valsartan and sacubitril/valsartan treatment resulted in a significant reduction of systolic BP; however, sacubitril/valsartan exhibited a superior antihypertensive effect compared to valsartan alone (40). Consistent with findings from earlier studies, treatment with sacubitril/valsartan reduced BP in a mice model of CRS, while the antihypertensive effect of valsartan was contingent upon dosage (41). Furthermore, administration of sacubitril/valsartan significantly attenuated systolic BP in both *db/db* and KKAy mice compared to vehicle (42). Thus, it can be inferred that the antihypertensive effects of ARNIs may contribute to the renoprotective effects in addition to the cardiovascular benefits.

**Table 1:** Effects of sacubitril/valsartan (LCZ696) on blood pressure in preclinical studies.

Animal models	Treatment period	Groups	Dose (mg/kg/day)	Blood pressure (mmHg)	Ref. no.
Streptozotocin-induced diabetic TGR (mREN2)27 rats	3 weeks	Valsartan	31	↓	(34)
		Sacubitril/valsartan	68	↓	
Subtotal nephrectomized rats	8 weeks	Valsartan	5	→	(35)
		Valsartan	15	↓	
		LCZ696	10	→	
		LCZ696	30	↓	
SHRSP rats	1, 2, 3, 4, 6 weeks	JD + valsartan	30	↓	(36)
		JD + sacubitril/valsartan	68	↓	
TGR (mREN2)27 rats	3 weeks	Thiorphan	0.1	–	(37)
		Irbesartan	15	↓	
		Irbesartan + thiorphan	15 + 0.1	↓	
		Irbesartan + thiorphan	15 + 1	↓	
Type 2 diabetic OLETF rats	24 weeks	Valsartan	30	↓	(38)
		Valsartan + hydralazine	30 + 3	↓	
		LCZ696	68	↓	
ZO rats	10 weeks	Sacubitril/valsartan (ZOSV)	68	↓	(39)
		Valsartan (ZOV)	31	↙	
		Hydralazine (ZOH)	30	↙	
Subtotal nephrectomized rats	8 weeks	CKD	–	↑	(40)
		LCZ696	60	↓	
		Valsartan	30	↓	
CRS mice	4 weeks	ANS-ARNI	60	↑	(41)
		ANS-VAL M	30	↓	
		ANS-VAL H	60	↓	
*db/db* mice	3 months	Valsartan	30	↓	(42)
		Sacubitril/valsartan	60	↓	
KKAy mice	3 months	Valsartan	30	↓	
		Sacubitril/valsartan	60	↓	

ANS, Chronic angiotensin II infusion, nephrectomy, and salt loading; CKD, Chronic kidney disease; CRS, Cardiorenal syndrome; JD, Japanese diet; OLETF, Otsuka Long Evans Tokushima Fatty; Ref. no., Reference number; SHRSP, Stroke-prone spontaneously hypertensive rats; ZO, Zucker obese; ↑, increased; ↓, decreased; ↙, tended to be decreased; →, not changed.

### Effects of sacubitril/valsartan on renal function and histology

#### Changes in serum and urinary biomarkers

Sacubitril/valsartan therapy markedly improved various renal injury biomarkers in multiple animal models of hypertension with kidney injury (Table 2). Both valsartan and sacubitril/valsartan resulted in a decrease in albuminuria in TGR (mREN2)27 rats as well as CRS, *db/db* and KKAy mice (34, 41, 42). Additionally, ARNI completely normalized both proteinuria and albuminuria while ARB only attenuated albuminuria, and these alterations occurred independently of blood pressure change in TGR (mREN2)27 rats with diabetic nephropathy (43). Nevertheless, the impact of dual blockade compared to control was more potent than that of single blockade (34). Polina et al. emphasized ARNI’s effect on reducing proteinuria in male Dahl salt-sensitive rats with renal disease and salt-sensitive hypertension (44). Sacubitril/valsartan also demonstrated greater creatinine clearances compared to the control group, and a similar pattern was observed compared to valsartan (34). However, there were no appreciable variations in plasma creatinine levels between the treatment groups (34, 41). Treatment with sacubitril/valsartan or valsartan diminished the level of kidney injury molecule (KIM)-1, neutrophil gelatinase associated lipocalin (NGAL) in various animal models including dogs and mice with CRS, *db/db* and KKAy mice, ZO rats and unilateral ureteral obstruction (UUO) rats (39, 42, 45–47). In addition, the level of clusterin, another urinary injury marker was reduced by both sacubitril and valsartan in ZO rats (39). Nevertheless, there is inconsistent information regarding cystatin C; which is known as a biomarker of GFR. A reduced level of plasma cystatin C correlates with the impairment of GFR and an elevated renal hyperfiltration. Plasma cystatin C levels were significantly increased in ZO rats following sacubitril/valsartan treatment (39). In contrast, a significant reduction in plasma cystatin C levels was reported in an experiment with CRS dogs following sacubitril/valsartan treatment (45). Renoprotective effects of both LCZ696 and valsartan have been demonstrated in several studies, including subtotal nephrectomy, JD fed SHRSP rats and ZO rats, where both drugs significantly attenuated urinary protein excretion (35, 36, 39). However, both valsartan and LCZ696 did not suppress the increase in serum creatinine in subtotal nephrectomized rats (35). Similarly, no significant differences were observed in plasma creatinine and urine albumin to creatinine ratio among the treatment groups (39) and these findings were supported in a study involving CRS rats treated with sacubitril/valsartan (48). Furthermore, our study demonstrated that LCZ696 markedly diminished proteinuria and attenuated the rise in blood urea nitrogen (BUN) and creatinine levels (38), suggesting a potential role of LCZ696 in preserving renal function. Additionally, other studies have shown a substantial reduction in plasma creatinine levels following treatment with sacubitril/valsartan (40, 49). In contrast to most of the study’s findings, a limited number of studies indicated that sacubitril/valsartan did not affect renal injury markers. Neither LCZ696 nor valsartan influenced renal function (serum creatinine, BUN, and cystatin C) among the experimental groups of UUO rats (46). The discrepancies in the outcomes of these studies may be attributed due to the variations in animal models, experimental methods, dosages of ARNI, and the limited sample size across different studies. Therefore, the data collectively indicates that, in the absence of cardiovascular diseases, ARNIs can improve kidney function across various animal models.

**Table 2:** Effects of sacubitril/valsartan (LCZ696) on renal function in preclinical studies.

Animal models	Treatment period	Groups	Dose (mg/kg/day)	Effects									Ref. no.
				Blood parameters					Urinary parameters				
				Serum creatinine (mg/dL)	Serum urea (mmol/L)	BUN (mg/dL)	Cystatin C (mg/L)	Creatinine clearance (mL/min)	Proteinuria/albuminuria (mg/day)	UPCR (mg/g)	KIM-1 (ng mgCr−1)	NGAL (pg/mL)	
ZO rats	10 weeks	Sacubitril/valsartan (ZOSV)	68	→	–	–	↑	–	↓	↓	↓	–	(39)
		Valsartan (ZOV)	31	→	–	–	↑	–	↓	↓	↓	–	
		Hydralazine (ZOH)	30	→	–	–	↑	–	↗	↗	→	–	
Subtotal nephrectomized rats	8 weeks	CKD	–	↑	–	↑	–	–	↑	↑	–	–	(40)
		LCZ696	60	↓	–	↓	–	–	↓	↓	–	–	
		Valsartan	30	→	–	→	–	–	↓	↓	–	–	
TGR (mREN2)27 rats	3 weeks	Thiorphan	0.1	↓	–	–	–	→	↓	–	–	–	(37)
		Irbesartan	15	↙	–	–	–	→	↓	–	–	–	
		Irbesartan + thiorphan	15 + 0.1	↓	–	–	–	→	↓	–	–	–	
		Irbesartan + thiorphan	15 + 1	→	–	–	–	↙	↓	–	–	–	
Streptozotocin induced diabetic TGR (mREN2)27 rats	3 weeks	Valsartan	31	↙	→	–	–	↓	↓	–	–	–	(34)
		Sacubitril/valsartan	68	↓	→	–	–	↑	↓	–	–	–	
Type 2 diabetic OLETF rats	24 weeks	Valsartan	30	↓	–	↓	–	–	↓	–	–	–	(38)
		Valsartan + hydralazine	30 + 3	↓	–	↙	–	–	↓	–	–	–	
		LCZ696	68	↓	–	↓	–	–	↓	–	–	–	
CRS mice	4 weeks	ANS-ARNI	60	→	–	–	–	→	↙	–	–	–	(41)
		ANS-VAL M	30	→	–	–	–	→	↓	–	–	–	
		ANS-VAL H	60	→	–	–	–	→	↓	–	–	–	
*db/db* mice	3 months	Valsartan	30	–	–	–	–	–	↓	–	↓	↙	(42)
		Sacubitril/valsartan	60	–	–	–	–	–	↓	–	↓	↓	
KKAy mice	3 months	Valsartan	30	–	–	–	–	–	↓	–	↓	↓	(42)
		Sacubitril/valsartan	60	–	–	–	–	–	↓	–	↓	↓	
CRS rat	7 days	Double IR	–	↑	–	↑	–	–	–	↑	–	–	(48)
		Double IR + levosimendan	30	→	–	→	–	–	–	→	–	–	
		Double IR + sacubitril/valsartan	10	→	–	→	–	–	–	→	–	–	
		Double IR + sacubitril/valsartan + levosimendan		→	–	→	–	–	–	→	–	–	
CRS dog	3 months	Sacubitril/valsartan	100	→	–	→	↓	–	–	–	↓	↓	(45)
CRS rat	63 days	CRS + High protein diet	–	↑	–	↑	–	–	–	↑	–	–	(53)
		CRS + high protein diet + Entresto (sacubitril/valsartan)	100	↓	–	↓	–	–	–	↓	–	–	
UUO rats	1 week	UUO	–	→	–	→	→	–	–	–	–	–	(46)
		UUO + LCZ696	68	→	–	→	→	–	–	–	–	–	
		UUO + valsartan	31	→	–	↙	↑	–	–	–	–	–	
		UUO + GS-444217	30; twice daily	↙	–	↑	↗	–	–	–	–	–	
Subtotal nephrectomized rats	8 weeks	Valsartan	5	→	–	–	–	–	↙	–	–	–	(35)
		Valsartan	15	→	–	–	–	–	↓	–	–	–	
		LCZ696	10	→	–	–	–	–	↙	–	–	–	
		LCZ696	30	↙	–	–	–	–	↓	–	–	–	
SHRSP rats	1, 2, 3, 4, 6 weeks	JD + valsartan	30	–	–	–	–	–	↓	–	–	–	(36)
		JD + sacubitril/valsartan	68	–	–	–	–	–	↓	–	–	–	
CRS mice	8 weeks	CRS	–	→	–	–	–	–	–	–	–	↑	(47)
		LCZ	60	→	–	–	–	–	–	–	–	↓	
		VAL	48	→	–	–	–	–	–	–	–	↓	

ANS, Chronic angiotensin II infusion, nephrectomy, and salt loading; BUN, Blood urea nitrogen; CKD, Chronic kidney disease; CRS, Cardiorenal syndrome; IR, Ischemia–reperfusion; JD, Japanese diet; KIM-1, Kidney injury molecule-1; NGAL, neutrophil gelatinase associated lipocalin; OLETF, Otsuka Long Evans Tokushima Fatty; Ref. No., reference number; SHRSP, Stroke-prone spontaneously hypertensive rats; UUO, Unilateral ureteral obstruction; JD, Japanese diet; ↑, increased; ↓, decreased; →, not changed; ↗, tended to be increased; ↙, tended to be decreased.

#### Effects on renal histological changes

ARNIs have been investigated for their impact on renal injury in various animal models mimicking CRS. Podocyte injury and apoptosis may lead to destruction of the glomerular filtration membrane which is associated with enhanced proteinuria in diabetic nephropathy (50). Sacubitril/valsartan treatment not only normalized podocyte foot process flattening (39) and improved podocyte density (42) but also more effectively prevented nephrin and podocin loss compared to valsartan monotherapy (39). The possible role of sacubitril/valsartan against protection of podocyte damage may be attributed to a reduction of glomerular transient receptor potential canonical (TRPC)-6 channels and an increase of renal Atrial natriuretic peptide (ANP), inducing inhibition of the nuclear factor of activated T cells (NFATc)-dependent regulator of calcineurin (Rcan)-1, (TRPC6-NFATc-Rcan1) pathway, while valsartan alone does not affect Rcan1 (34, 51). In a streptozotocin- induced diabetic TGR (mREN2)27 rat model, sacubitril/valsartan significantly reduced focal segmental glomerulosclerosis (FSGS) and glomerulosclerosis index (GSI) while maintaining podocyte integrity, indicating improved renal tissue protection (34). Combination of NEPI and ARB also decreased glomerular and tubulointerstitial fibrosis (46, 52). However, the combination therapy demonstrated a more pronounced alleviation in fibrosis compared to single therapy with valsartan (46). Similarly, improvement of renal fibrosis was observed in different studies conducted in mice, dogs, and rats with CRS after receiving both sacubitril and valsartan (45, 47, 48, 53, 54). In contrast, LCZ696 in CRS mice demonstrated a more marked attenuation in the percentage of fibrosis than valsartan (47). Rat models of subtotal nephrectomy revealed severe glomerulosclerosis, tubulointerstitial injury, dilatation of tubules, and widening of the interstitium, while administration of LCZ696 resulted in a substantial decrease in both glomerulosclerosis and tubulointerstitial scores (35, 40). It is noteworthy that only valsartan significantly reduced the tubulointerstitial score, while it did not significantly affect the glomerulosclerosis scores. Furthermore, LCZ696 exhibited more prominent and substantial effects in inhibiting the progression of glomerulosclerosis in comparison to valsartan (35). Whereas, LCZ treatment demonstrated a greater degree of improvement in both of these parameters compared with valsartan alone (40). Significant attenuation of glomerulosclerosis and tubular damage by treatment with valsartan and sacubitril/valsartan was confirmed in a number of previous studies with SHRSP rats, type 2 diabetic OLETF rats and streptozotocin induced diabetic rats (36, 38, 49). In line with previous findings, Habibi et al. demonstrated a reduction in interstitial fibrosis following treatment with sacubitril/valsartan (39). Furthermore, administration of sacubitril/valsartan reduced glomerulosclerosis in type-2 diabetic *db/db* and KKAy mice, as indicated by decreased levels of fibronectin and collagen type IV expression (42). Taken together, sacubitril/valsartan shows potential protective effects against renal injury in diverse animal models of CRS.

#### Effects on renal inflammation and oxidative stress

**Figure 1:**
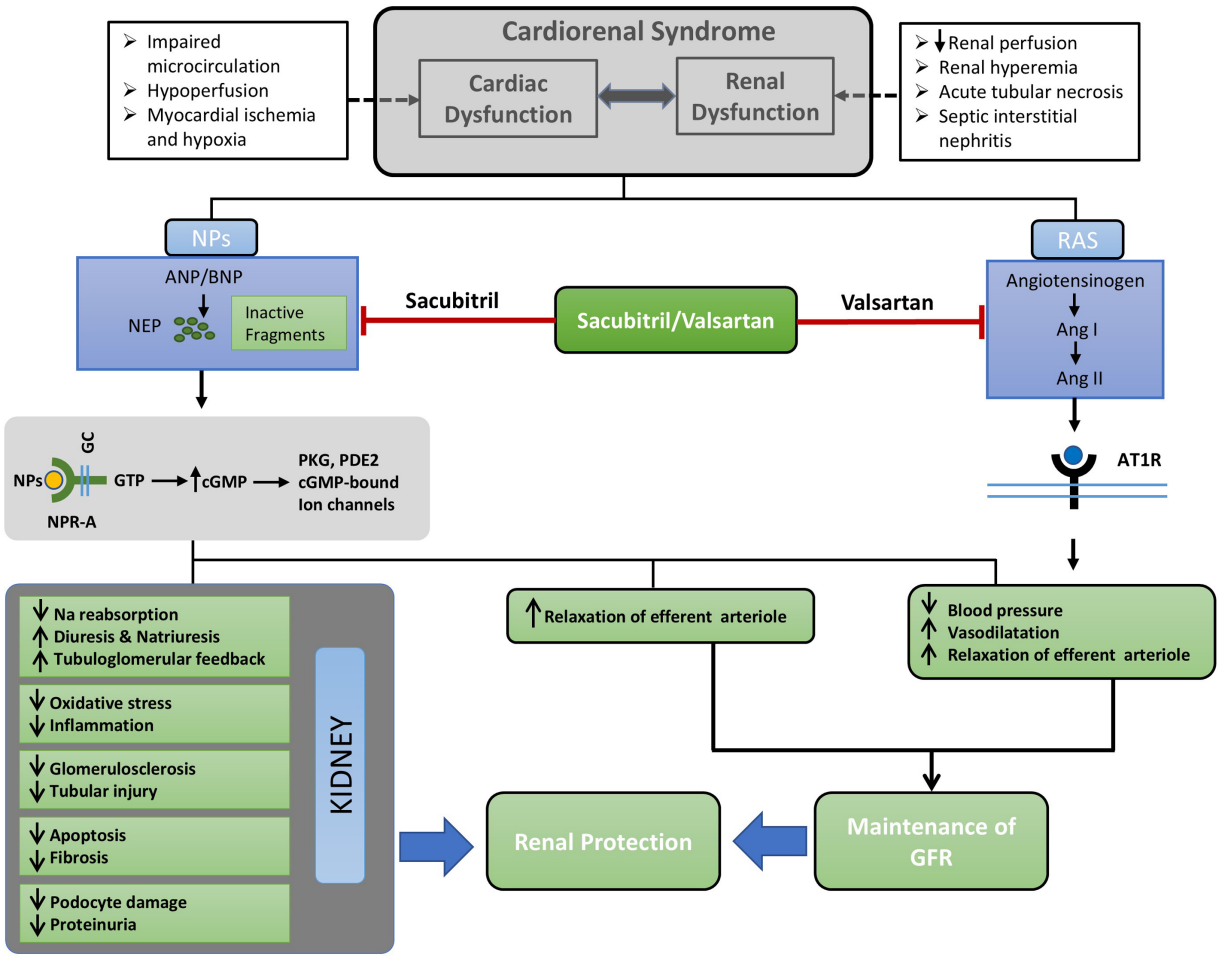
Concise outline of the possible mechanisms underlying the renoprotective benefits of ARNI (Sacubitril/Valsartan). Cardiorenal syndrome may stimulate the renin-angiotensin system, resulting in the overproduction of natriuretic peptides (NPs). Sacubitril/valsartan inhibits neprilysin (NEP) and the binding of Ang II to its receptor AT1R; thereby preventing the activation of intracellular signaling cascades. Consequently, there is a reduction of blood pressure and sodium reabsorption, vasodilatation, and relaxation of afferent arteriole. These alterations in glomerular hemodynamics are caused by dual blockade with sacubitril/valsartan result in the maintenance of GFR. On the other hand, increased levels NPs as a result of inhibition of NEP by sacubitril alleviates renal tissue injury by reducing oxidative stress, inflammation, podocyte injury, glomerulosclerosis, and fibrosis. Ultimately, the simultaneous improvement of renal tissue injury alongside the maintenance of GFR results in renal protection facilitated by sacubitril/valsartan. Ang I, Angiotensin I; Ang II, Angiotensin II; ANP, Atrial natriuretic peptide; AT1R, Angiotensin type1 receptor; BNP, Brain natriuretic peptide; cGMP, Cyclic guanosine monophosphate; GC, Guanylyl cyclase; GFR, Glomerular filtration rate; GTP, Guanosine triphosphate; NEP, Neutral endopeptidase; NPs, Natriuretic peptides; NPR-A, Natriuretic peptide receptor A; PDE2, Phosphodiesterase2; PKG, Protein kinase G; RAS, Renin angiotensin system.

Renal inflammation and fibrosis are linked to the overexpression of several inflammatory genes in the kidney tissues. The diabetic TGR (mREN2)27 rat model showed a significant reduction in the gene expression of the macrophage marker CD68 following treatment with sacubitril/valsartan (34). Further investigations using LCZ696 or valsartan demonstrated a noticeable decrease in the levels of inflammatory mediators. Ding et al. observed a significant suppression of renal proinflammatory cytokines such as pro-interleukin (pro-IL)-1β, pro-IL-18, NLR family pyrin domain containing (NLRP)-3 and tumor necrosis factor (TNF)-α following treatment with LCZ696 (46). In contrast to valsartan, LCZ696 treatment significantly reduced renal inflammation in UUO rats. Oxidative stress is associated with an imbalance in the regulation of antioxidant and oxidant enzymes, which can lead to inflammation. Thus, it is possible that UUO causes oxidative stress, which in turn causes inflammation. LCZ696 treatment reduced oxidative stress by upregulating manganese superoxide dismutase (MnSOD) and thioredoxin expression and by downregulating inducible nitric oxide synthase (iNOS) and thioredoxin-interacting protein (TXNIP) expression. Moreover, LCZ696 reduced the excretion of 8-Hydoxy-2′-deoxyguanosine (OHdG) in the urine (46). Furthermore, a decrease in the activity of antioxidant enzymes is a hallmark of tissue damage. Accordingly, LCZ696 was observed to restore the activity of anti-oxidant enzymes, including superoxide dismutase (SOD), catalase (CAT), glutathione peroxidase (GPx), and glutathione-S-transferase (GST), in streptozotocin-induced diabetic rats (49), suggesting protection of renal tissues from oxidative stress. On top of that, expressions of NADPH oxidase (NOX)-4, gp91phox, p22phox and oxidized protein play a key role in oxidative stress which were upregulated in high protein fed CRS rats. Sacubitril/valsartan treatment decreased the expression of these protein in CRS rats (53). In addition, the levels of other markers of oxidative stress, such as 4-hydroxynonenal (HNE) and NOX4, were reduced by treatment with sacubitril/valsartan in diabetic *db/db* and KKAy mice. Besides, expression of antioxidative regulator; the nuclear factor erythroid 2-related factor (Nrf)-2 and antioxidant enzyme SOD were enhanced by both sacubitril/valsartan and valsartan in *db/db* and KKAy mice (42). Conversely, a study conducted on streptozotocin-induced diabetic rats, treated with either LCZ696 or valsartan for 6 weeks showed a reduction in levels of inflammatory markers TNF-α, interleukin (IL)-1β, and IL-6 in the kidney and blood, as well as an increase in levels of the anti-inflammatory cytokine IL-10 (49). Nuclear factor kappa-light-chain-enhancer of activated B cells (NF-Κβ) p65 expression level, is used as a marker for tissue inflammation. As anticipated, JD significantly increased inflammation in the tissues, as evidenced by the increased expression of NF-Kβ in SHRSP rats. Nevertheless, the presence of inflammation was considerably reduced by both sacubitril/valsartan and valsartan (36). Consistent with these results, both LCZ696 and valsartan decreased NF-Kβ activation and reduced the levels of monocyte chemoattractant protein (MCP)-1, iNOS, and cyclooxygenase (COX)-2, while also enhancing the Nrf-2 antioxidant pathway in rats that undergone subtotal nephrectomy (40). Importantly, the activation of cyclic guanosine monophosphate-adenosine monophosphate (cGMP-AMP) synthase-stimulator of interferon genes (cGAS-STING) signaling by self-DNA is linked to inflammation and is observed in diabetic kidney disease. This activation was found to be suppressed by sacubitril/valsartan and valsartan treatment (42). Additionally, 3-nitrotyrosine (NTY) immunostaining is often used as a marker for nitroso-oxidative stress in glomeruli, proximal and distal tubules. Notably, administration of sacubitril/valsartan effectively suppressed the increase in intensity of 3-NTY staining by 34% in the glomeruli. A comparable pattern was noted in the staining intensity of 3-NTY in the tubular region among the animals treated with sacubitril/valsartan. Besides, expression of NOX-4 in kidney was significantly attenuated in animals treated with sacubitril/valsartan (39). Activation of inflammatory and oxidative pathway in CKD might lead to renal fibrosis which is manifested by upregulation of plasminogen activator inhibitor (PAI)-1, transforming growth factor (TGF)-β, connective tissue growth factor (CTGF) and α-smooth muscle actin (SMA) and changes in these markers were mitigated by both LCZ696 and valsartan (40, 46). These findings indicate a potential role of sacubitril/valsartan in protecting renal functions by inhibiting renal inflammation, fibrosis, oxidative stress. These renoprotective effects may be achieved through various mechanisms (Figure 1), including the inhibition of TGF-β1/Smad 2 or 3/CTGF/Collagen IV (47) or ASK1/JNK/p38 MAPK (46) or TRPC6-NFATc-*Rcan1* (34), as well as the activation of the Nrf-2 pathway (40).

### Pharmacological effects of ARNIs on renovascular outcomes: evidence from clinical studies

ARNIs, particularly sacubitril/valsartan has been approved for the treatment of HF patients with reduced ejection fraction (55). The PARADIGM-HF (Prospective Comparison of ARNI with ACE inhibition to Determine Impact on Global Mortality and Morbidity in Heart Failure) trial found no difference in the expected kidney composite outcome between the sacubitril/valsartan and enalapril groups (3). However, the sacubitril/valsartan group had a 37% lower risk of new composite kidney outcomes added during post-hoc analysis. Additionally, sacubitril/valsartan treatment was associated with an increased risk of albuminuria when compared to enalapril, and urinary albumin creatinine ratio (UACR) was markedly elevated in the sacubitril/valsartan group following 1 and 8 months cohort, while the rate of decline in eGFR was comparatively reduced. Although the primary focus of the PROVE-HF (Effects of Sacubitril/Valsartan Therapy on Biomarkers, Myocardial Remodeling and Outcomes) trial was on HF, a secondary analysis revealed potential enhancements in kidney function following treatment with ARNIs (4). The PARAMOUNT (Prospective Comparison of ARNI With ARB on Management of Heart Failure With Preserved Ejection Fraction) trial also found that sacubitril/valsartan compared to valsartan treatment for 9 months increased albuminuria in patients with preserved ejection fraction, despite the former having greater antihypertensive effect and a slower deterioration of eGFR (5). Prior studies indicated that ARNI greatly improved HF outcomes and lowered systolic BP more effectively than valsartan; however, it is crucial to determine whether the renoprotective effect of ARNI is independent of the drug’s beneficial effects on hemodynamics and HF (56, 57). Additionally, the PARAGON-HF (Prospective Comparison of ARNI With ARB Global Outcomes in HF With Preserved Ejection Fraction) trial found that sacubitril/valsartan reduced the risk of composite renal events and associated with decrease in eGFR compared to valsartan (58). However, there was no difference in the risk of progression to end stage kidney diseases (ESKD). The UK HARP-III (United Kingdom Heart and Renal Protection) trial evaluating 414 patients with glomerular filtration rate (GFR) 20–60 mL/min/1.73 m^2^ found no significant difference in primary outcomes of measured GFR between sacubitril/valsartan and irbesartan (59). However, sacubitril/valsartan was associated with a nonsignificant 9% reduction in UACR compared to irbesartan, which was associated with a reduction in BP. Conversely, limited studies on HF patients exhibited an elevation in proteinuria (60). The discrepancies in trial outcomes concerning albuminuria may be attributed to various factors (including the basal level of albuminuria or severity of kidney injury) that affect the progression of kidney disease in populations with proteinuric CKD compared to those with HF. Prior observations of an elevation in albuminuria, despite a deceleration in the decline of eGFR induced by ARNIs, may be attributable to these potential explanations. Inhibition of neprilysin increases the bioavailability of NPs; simultaneous inhibition of AT1 receptor and neprilysin further decreases systemic BP, resulting in selective relaxation of the preglomerular afferent arteriole and relative constriction of the efferent arteriole. This may lead to increased intracapillary hydraulic pressure despite a reduced renal perfusion pressure, which may subsequently elevate the filtration fraction and maintain GFR under reduced systemic BP (61, 62). The increased intracapillary hydraulic pressure, coupled with the direct influence of ARNIs on the glomerular barrier may lead to an increase in albumin ultrafiltration, which in combination with possible decrease in tubular protein reabsorption could lead to a modest increase in albuminuria (3, 5). Randomized clinical trials evaluating the renal outcomes of sacubitril/valsartan are summarized in Table 3.

**Table 3:** Effects of sacubitril/valsartan (LCZ696) on renal outcomes in clinical trials.

Trial’s name	Comparator	Subgroup	No.	Population	Definition of renal events	Rate of renal events		HR (95% CI)	eGFR (mL/min/1.73 m^2^)						Ref.
						Sac/val	Comparator		Sac/val			Comparator			
									Baseline	Reduction (End of trial)	Reduction/year	Baseline	Reduction (End of trial)	Reduction/year	
PARADIGM-HF post-hoc analysis	Enalapril	All	8,399	Chronic HFrEF, LVEF≤40%	↓ eGFR≥50% ESRD	0.9	1.4	0.63 (0.42–0.95)	70 ± 20	−7.8	−1.61^†^	70 ± 20	−10.2	−2.04	(3)
		30 < eGFR<60	3,061			1.2	1.8	0.64 (0.34–1.19)			−1.98^§^			−2.29	
		eGFR≥60	5,388			0.7	1.1	0.63 (0.36–1.01)			−0.80^†^			−1.55	
PARAGON-HF	Valsartan	All	4,796	Chronic HFpEF, LVEF≥45%	↓ eGFR≥50% ESRD Renal death	1.4	2.7	0.50 (0.33–0.77)	63 ± 19	−7.7^‡^	−2.0^†^	62 ± 19	−10.1^‡^	−2.7	(58)
		30 < eGFR<60	2,341			1.4	2.7	0.50 (0.28–0.92)							
		eGFR≥60	2,454			1.4	2.6	0.51 (0.29–0.93)							
PARAMOUNT-HF	Valsartan	All	301	Chronic HFpEF, LVEF≥45%	↑ Serum creatinine >0.3 g/dL and >25%	12	18	NA	66.5 ± 19	−1.5^*^	−2.2^‡^	64.3 ± 21	−5.2	−7.5^‡^	(5)
PIONEER-HF	Enalapril	All	881	ADHF, LVEF≤40%	↑ Serum creatinine ≥0.5 g/dL, ↓ eGFR≥25%	13.6	14.7	0.93 (0.67–1.28)							(70)
UK HARP-III	Irbesartan	All	414	CKD, eGFR 20-60	↓ eGFR≥25%	34	32	NA	35	5.2		36	6.1		(59)

ADHF, acute decompensated heart failure; CI, confidence interval; CKD, chronic kidney disease; eGFR, estimated glomerular filtration rate (in mL/min/1.73 m^2^); ESRD, end-stage renal disease; HFpEF, heart failure with preserved ejection fraction; HFrEF, heart failure with reduced ejection fraction; HR, hazard ratio; LVEF, left ventricular ejection fraction; NA, not available; PARADIGM-HF, Prospective Comparison of ARNI with ACEI to Determine Impact on Global Mortality and Morbidity in Heart Failure; PARAGON-HF, Prospective Comparison of ARNI with ARB Global Outcomes in HF with Preserved Ejection Fraction; PARAMOUNT, Prospective Comparison of ARNI with ARB on Management of Heart Failure with Preserved Ejection Fraction; PIONEER-HF, Patients Stabilized from an Acute Heart Failure Episode; UK HARP-III, United Kingdom Heart and Renal Protection-III.

^*^p = 0.002 vs. comparator; ^†^*p* < 0.001 vs. comparator; ^§^*p* < 0.05; ^‡^calculated.

Insights into the positive renal outcomes of ARNI in patients with concomitant heart failure with reduced ejection fraction (HFrEF) and CKD have been demonstrated in a number of real-world studies (63–68). Improvement of eGFR was observed in a study of 108 patients with HFrEF treated with sacubitril/valsartan compared to those managed with standard HF care without ARNI (73.8 vs. 61.2 mL/min/1.73 m^2^, *p* < 0.001). Significant improvement in left ventricular ejection fraction (LVEF) was also found with sacubitril/valsartan (42.4% vs. 34.2%, *p* < 0.05) (68). Similarly, in another real-world study conducted in 54 consecutive outpatients with HFrEF (53.7% had CKD at baseline), renal function improved during a follow-up period of 12-months compared to historical controls who received standard medical care (65). Additionally, sacubitril/valsartan was more effective in reducing CV deaths or hospitalizations than standard HF therapy in patients with significant renal insufficiency at baseline in a study of 932 patients with HFrEF (63). Martínez-Esteban et al. also reported beneficial role of sacubitril/valsartan in patients suffering from advanced CKD and HFrEF as evidenced by improvement in eGFR (67). Outcomes of sacubitril/valsartan (LCZ696) in CKD and ESRD patients in real-world observational studies are enlisted in Table 4.

**Table 4:** Outcomes of sacubitril/valsartan (LCZ696) in CKD and ESRD patients in real-world observational studies.

Ref.	eGFR (mL/min/1.73 m^2^)	*n*	Age (years)	Follow-up	Population type	Hypertension (years)	LVEF (%)	Outcomes	Adverse events
Chang et al. (63)	<30	102	61.3 ± 14.5	15 months	HFrEF with CKD	NR	27.0 ± 6.8	28% fewer CVD or HF hospitalization	NR
	30–60	262						14% fewer CVD or HF hospitalization	
Ito et al. (56)	≥15 to <30	7	65.8 ± 9.1	8 weeks	Hypertension with CKD	9.4 ± 6.4	NR	17.7/5.5 mm Hg reduction for mSBP/mDBP	4 (57.1%)
	≥30 to <60	25						21.3/9.1 mmHg reduction for mSBP/mDBP	10 (40%)
Lee et al. (71)	<15	23	67 ± 9.0	132 days	HFrEF with CKD	NR	29.7 ± 2.4	hsTnT and sST2 were reduced, and LVEF was also improved	5 (21.7%)
Martínez-Esteban et al. (67)	29.4 ± 8.3	25	73.2 ± 5.9	31 months	HFrEF with CKD	NR	36.4 ± 8.9	Improvement of eGFR and LVEF	NR
Jia et al. (72)	43.14 ± 19.21 (Sacubitril/valsartan)	198	70.45 ± 13.99	12 weeks	HFpEF with CKD	NR	52.00	Attenuation of decline in renal function and reversal of myocardial remodeling	NR
	49.87 ± 17.99 (ACEi/ARB)	198	70.56 ± 14.76				54.00		

CKD, chronic kidney disease; ESRD, end-stage renal disease; eGFR, estimated glomerular filtration rate; LVEF, left ventricular ejection fraction; HFrEF, heart failure with reduced ejection fraction; HFpEF, heart failure with preserved ejection fraction NR, not reported; CVD, cardiovascular deaths; HF, heart failure; mSDP, mean systolic blood pressure; mDBP, mean diastolic blood pressure; hsTnT, high-sensitive troponin T; sST2, soluble ST2.

A meta-analysis of 3,460 individuals with HF and CKD found that ARNI treatment significantly increased eGFR and decreased systolic BP, diastolic BP, and N-terminal prohormone of brain natriuretic peptide (NT-proBNP) compared to ACEi or ARB (69). However, no difference was found in UACR. In a recent meta-analysis, Feng et al. examined 11 studies involving 21,716 patients to assess the renal safety and effectiveness of ARNIs. The analysis revealed that ARNIs had a positive impact on renal outcomes by reducing renal dysfunction and increasing eGFR, without any significant increase in the risk of hyperkalemia (6). ARNIs are thought to provide renoprotective advantages in HF patients by improving renal blood flow, leading to increased pressure in the glomerulus and a rise in the GFR (7). Collectively, recent evidence strongly indicates that ARNIs are more effective than RAS inhibitors in promoting renoprotection in patients with CKD and HF by reducing blood pressure and decreasing albuminuria. Evidence regarding renoprotective effects in patients with advanced kidney diseases with high levels of albuminuria, however, is limited.

## Conclusion

Currently, there is no available drug for standard medical care of patients suffering from CRS due to its complex pathogenesis. Since RAS and NPs play a crucial role in regulating renal and cardiovascular pathogenesis, the inhibition of RAS and NP by sacubitril/valsartan may offer positive cardiorenal outcome in CRS. Accumulating data suggests that ARNIs have renoprotective effect through decelerating the decline in eGFR and cardiovascular protection by enhancing the LVEF in HF patients within the context of CRS. As the drug is being increasingly used in clinical practice, additional experiments are required to unravel the complex mechanisms of ARNI in modifying CRS pathophysiology and to evaluate potential cardiorenal protection that have been reported. Moreover, investigation is required to ascertain the long-term benefits of ARNIs, particularly in populations with varying degrees of renal impairment (different subtypes of CRS) and across different heart failure phenotypes that closely resemble the CRS conditions to better understand the enduring renal outcomes.
